# Identification of Potential Vectors and Detection of Rift Valley Fever Virus in Mosquitoes Collected Before and During the 2022 Outbreak in Rwanda

**DOI:** 10.3390/pathogens14010047

**Published:** 2025-01-08

**Authors:** Isidore Nsengimana, Emmanuel Hakizimana, Jackie Mupfasoni, Jean Nepomuscene Hakizimana, Augustino A. Chengula, Christopher J. Kasanga, Gillian Eastwood

**Affiliations:** 1 Department of Veterinary Microbiology, Parasitology and Biotechnology, Sokoine University of Agriculture, Morogoro P.O. Box 3000, Tanzania; 2 Rwanda Inspectorate, Competition and Consumer Protection Authority, Kigali P.O. Box 375, Rwanda; 3 SACIDS Africa Centre of Excellence for Infectious Diseases, SACIDS Foundation for One Health, Sokoine University of Agriculture, Morogoro P.O. Box 3297, Tanzania; 4 Rwanda Biomedical Center (RBC), Ministry of Health, Kigali, P.O. Box 7162, Rwanda; 5 Department of Entomology; The Global Change Center at Virginia Tech; and the Center for Emerging Zoonotic & Arthropod-Borne Pathogens (CeZAP), Virginia Polytechnic Institute and State University, Blacksburg, VA 24061, USA

**Keywords:** Rift Valley fever, mosquitoes, vectors, RVFV detection, outbreak, Rwanda

## Abstract

Rift Valley fever virus (RVFV) is an emerging mosquito-borne arbovirus of One Health importance that caused two large outbreaks in Rwanda in 2018 and 2022. Information on vector species with a role in RVFV eco-epidemiology in Rwanda is scarce. Here we sought to identify potential mosquito vectors of RVFV in Rwanda, their distribution and abundance, as well as their infection status. Since an outbreak of RVF occurred during the study period, data were obtained both during an interepidemic period and during the 2022 Rwanda RVF outbreak. Five districts of the eastern province of Rwanda were prospected using a combination of unbaited light traps and Biogents (BG Sentinel and Pro) traps baited with an artificial human scent during three periods, namely mid-August to mid-September 2021, December 2021, and April to May 2022. Trapped mosquitoes were morphologically identified and tested for viral evidence using both RT-PCR and virus isolation methods on a Vero cell line. A total of 14,815 adult mosquitoes belonging to five genera and at least 17 species were collected and tested as 765 monospecific pools. *Culex quinquefasciatus* was the most predominant species representing 72.7% of total counts. Of 527 mosquito pools collected before the 2022 outbreak, a single pool of *Cx. quinquefasciatus* showed evidence of RVFV RNA. Of 238 pools collected during the outbreak, RVFV was detected molecularly from five pools (two pools of *Cx. quinquefasciatus*, two pools of *Anopheles ziemanni*, and one pool of *Anopheles gambiae* sensu lato), and RVFV was isolated from the two pools of *Cx. quinquefasciatus*, from Kayonza and Rwamagana districts, respectively. Minimum infection rates (per 1000 mosquitoes) of 0.4 before the outbreak and 0.6–7 during the outbreak were noted. Maximum-likelihood phylogenetic analysis indicates that RVFV detected in these mosquitoes is closely related to viral strains that circulated in livestock in Rwanda and in Burundi during the same RVF outbreak in 2022. The findings reveal initial evidence for the incrimination of several mosquito species in the transmission of RVFV in Rwanda and highlight the need for more studies to understand the role of each species in supporting the spread and persistence of RVFV in the country.

## 1. Introduction

Rift Valley fever virus (RVFV) (genus *Phlebovirus*, family *Phenuiviridae*, order Hareavirales, class Bunyaviricetes) is an emerging arbovirus endemic to Africa and the Arabian Peninsula [1]. The associated disease, Rift Valley fever (RVF), was first described in a Merino sheep farm in the Rift Valley region of Kenya during an excessive rainfall season in 1930 [2] and has since been shown to circulate widely in Africa and the Arabian Peninsula. The disease is of One Health concern and affects ruminant livestock such as cattle, goat, sheep, and camels, and can also cause severe disease in humans [3] The infection in livestock is transmitted by mosquitoes and characterized by abortion storm and high mortality in young animals [4]. Humans can also gain infection via the bite of an infected mosquito, or through contact with infected animals, their blood or tissues [3]. Although most clinical infections in people are asymptomatic or produce a mild feverish syndrome that is self-limiting, severe complications with meningo-encephalitis, retinitis, and hemorrhagic syndrome can sometimes occur [5].

RVF is a climate-sensitive disease, which has caused devastating recurrent outbreaks with severe impact on rural livelihoods, especially in sub-Saharan Africa [6,7,8,9]. RVFV epizootics/epidemics have been associated with periods of unusually heavy and persistent rains causing widespread flooding, followed by massive mosquito breeding [10,11,12]. In East Africa, a connection between these weather extremes with a warm phase of El Niño Southern Oscillation (ENSO) has been demonstrated [13,14]. Climatic changes impact the vector habitats and distribution, and consequently influence the emergence of new risk areas and outbreak patterns [15,16,17,18]. Understanding the role of mosquito species in the transmission of RVFV in each geographical region is thus fundamental for guiding the prevention and control of RVF outbreaks [19]. Although there is still a knowledge gap about RVFV maintenance in the environment [20,21], mosquitoes have been reported to sustain RVFV persistence between outbreaks [19,22]. Floodwater mosquitoes, namely species of the genus *Aedes*, classified as RVFV primary vectors, deposit transovarially infected eggs that can survive long periods in the dried mud substrate [19,22]. During subsequent precipitation flooding, such eggs hatch and develop into adult mosquitoes that transmit the virus to nearby amplifying vertebrate hosts. Secondary mosquito vectors, namely *Culex*, *Anopheles*, and *Mansonia* species, which breed in these flooded areas, feed on RVFV-infected animals and further spread the virus to additional vertebrate hosts, thus spreading the virus longer distances [23]. Unapparent low-level circulation of the virus between mosquitoes and vertebrate hosts is another maintenance mechanism and likely the most predominant in certain tropical ecosystems with year-round mosquito activity [24].

According to the literature, more than 50 mosquito species have been shown to be associated with RVFV natural infection in the field, although their infection frequency varies significantly [21,23,25,26]. A comprehensive entomological investigation of the largest 2006/7 RVFV outbreak in Kenya revealed 10 different mosquito species implicated in the transmission of RVFV, with *Aedes mcintoshi* and *Aedes ochraceus* being the major vectors, alongside other secondary vectors such as *Mansonia uniformis*, *Mansonia africana*, *Culex pipens pipiens*, *Culex poicilipes*, *Culex univitattus*, *Culex pipiens quinquefasciatus*, and *Culex annulioris* [12,27]. In West Africa, *Aedes. ochraceus*, *Aedes vexans*, and *Aedes dalzieli* were incriminated in the enzootic transmission of RVFV [28], while *Culex poicilipes* was consistently reported as a major outbreak vector in Senegal and Mauritania [29]. *Culex pipiens*, a sibling of *Culex quinquefasciatus*, was the principal vector in the extensive 1977/8 Egyptian outbreak [30], whereas *Culex tritaeniorhynchus* and *Aedes vexans* were incriminated in the 2000 RVF outbreak in Saudi Arabia [31]. In Madagascar, ten mosquito species were found to be naturally infected during a 2021 outbreak, with *Aedes* species and *Anopheles gambiae* sensu lato added to the list [26]. Besides the history of natural infection with RVFV, two further criteria used to assess the vector status of mosquito species include (1) their vector competence, or laboratory-demonstrated ability to support virus infection, dissemination, and transmission to susceptible hosts, and (2) vector–host associations, as demonstrated by the feeding behavior. According to the classification proposed by Tantely et al. [25], species may be categorized as a potential vector, candidate vector, or vector of RVFV, if one, two, or three criteria are validated, respectively.

Although Rwanda is situated in the Rift Valley region [32], a known endemic area of RVFV, and has, over the last two decades, made substantial progress in developing the livestock sector, in particular, rebuilding the cattle herd after it was decimated during the 1994 Tutsi Genocide [33], there is little information on the epidemiology of RVFV in the country. Rwanda’s climate and landscape, with hills and marshy bottoms mainly shaped by the Akagera River and its tributaries draining the hot and dry lowlands of the east and central plateaus, offer appropriate conditions for mosquito activity. In addition, the country’s recent expansion of rice-irrigated farming [34] coupled with the increase in “improved cattle” population [35] (such cattle being kept outside overnight and being a host available for mosquitoes) contribute to the country’s vulnerability to RVFV [36,37].

**Figure 1:**
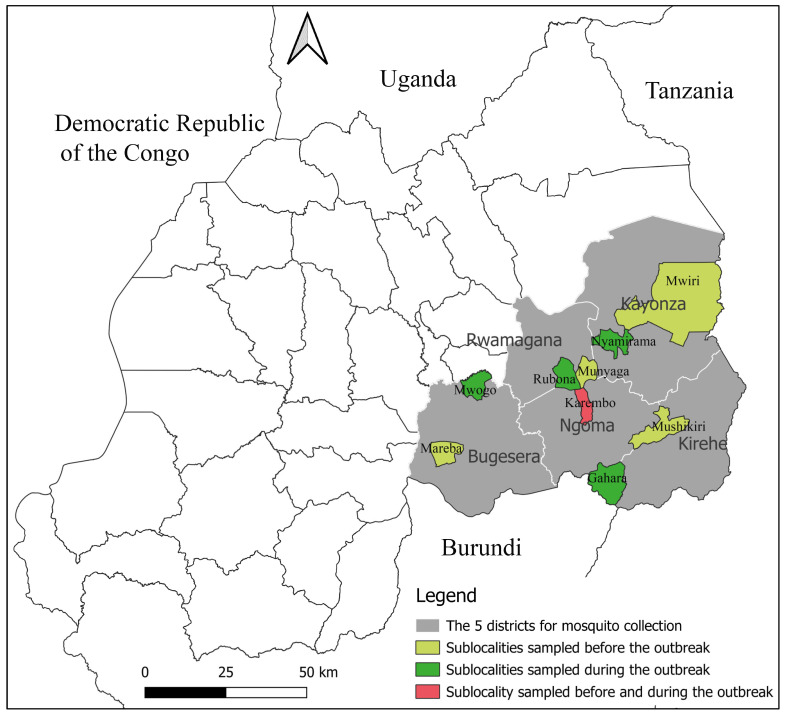
Map of Rwanda showing the five districts of the study area, and location of sampling sites used before and/or during the outbreak. The map was drawn using the QGIS version 3.24.1 freely available at https://www.qgis.org/en/site/https://www.qgis.org/en/site/, accessed on 25 October 2024.

RVFV was confirmed for the first time in Rwanda’s livestock in 2012 in Bugesera district (Figure 1), eastern province [38]. After a series of localized small-scale outbreaks occurring almost annually, two large epizootics occurred in 2018 [39] and 2022 [40], with both epicenters identified within the eastern province of the country. Despite these outbreak reports clearly showing RVFV circulation and persistence in the region, no assessment of the mosquito species involved in the RVFV transmission and spread of this zoonosis has taken place, and few entomological investigations of mosquito species have been conducted in Rwanda (outside malaria surveillance). The identification of potential RVFV vectors and determination of their abundance, distribution, and infection rates is critical for the assessment of disease transmission risk and outbreak prediction [41]. The purpose of this study, therefore, was to identify mosquito species in the region that could serve as vectors of RVFV, and determine their abundance, distribution, and infection status in the eastern province of Rwanda.

## 2. Materials and Methods

### 2.1. Study Area and Design

A repeated cross-sectional survey of adult mosquitoes was conducted during three collection periods from mid-August 2021 to May 2022 in five districts (Kirehe, Ngoma, Kayonza, Rwamagana, and Bugesera) of the eastern province of Rwanda (Figure 1). The eastern province is a dry and hot lowland zone rich in lakes and marshlands [32] and has been the region most affected by RVF outbreaks [39]. The area shares borders with Burundi in the south, Tanzania in the east and Uganda in the north. Alongside the Akagera River, this province hosts “Akagera National Park”, which is a vast grassland harboring wild ruminants such as buffaloes and many antelope species [42]. The average temperature in this eastern plateau ecosystem varies between 20 and 22 °C and the mean annual rainfall ranges between 700 and 1100 mm. The rainfall is bimodal; a short rainy season generally spans from mid-September to December, with a long rainy season from March to May [32]. In each of the five study districts, one collection site with a history of RVF outbreak was selected for mosquito collection.

### 2.2. Mosquito Collection and Identification

Adult mosquitoes were trapped at selected sites during three periods, mid-August to mid-September 2021 (period 1), December 2021 (period 2), and April to May 2022 (period 3), with the latter coinciding with the 2022 Rwanda RVF outbreak. Mosquito collections used a combination of unbaited CDC light traps (John W. Hock, Gainesville, FL, USA), and BG Sentinel or BG Pro traps (Biogents AG, Regensburg, Germany) baited with both BG lure (a Biogents-supplied mosquito attractant mimicking a human skin scent) and CO_2_ (as produced by a sugar-yeast blend) [43]. During each trapping session, 9 traps (3 light traps, 4 BG Pro, and 2 BG Sentinel) were deployed at each site, placed near cattle kraal in a farm or inhabited area. Traps were set at least 100 m apart from one another and operated from 15:00 h for Biogents traps or 18:00 h for CDC light traps and retrieved at 07:00 h of the next morning for two consecutive days. Trapped mosquitoes were transported to the nearby sorting site, anesthetized using 99.5% triethylamine (Sigma-Aldrich, St. Louis, MO, USA) for 3 min, sorted by sex, and identified by species using the morphological identification keys [44,45,46]. Female mosquitoes were pooled by species, location, and date (up to 20 mosquitoes per pool) and transported in liquid nitrogen to the Rwandan National Veterinary Laboratory, Virology unit for storage at −80 °C. For virus screening, mosquitoes were shipped on dry ice to Virginia Polytechnic Institute and State University, VA, USA and stored at −80 °C until processed.

### 2.3. Mosquito Homogenization

Subsequent mosquito processing was performed in a biosafety level 3 (BSL-3) containment facility. Each mosquito pool was transferred into a 2 mL vial containing a metal ball-bearing and 600 mL of mosquito diluent (Dulbecco’s Modified Eagle Medium (DMEM) supplemented with 20% fetal bovine serum (FBS), 0.37% sodium bicarbonate, 1% antibiotic (penicillin-streptomycin), and 1% antimycotic (Amphotericin B)). Mosquitoes were homogenized at 30 Hz/s for 2 min using Tissuelyser II (Qiagen, Hilden, Germany) before centrifugation at 10,000 rpm for 4 min at 4 °C to clarify the supernatant.

### 2.4. Viral RNA Extraction and RVFV Detection by RT-PCR

Viral RNA was extracted from the supernatant of each mosquito pool using a QIAamp viral RNA mini kit (Qiagen) according to the manufacturer instructions. Reverse transcription of RNA into cDNA as well as conventional PCR amplification of a target RVFV genome fragment were performed using a One-Step RT-PCR kit (Qiagen), according to the manufacturer instructions. RVFV-specific primers proposed by Ibrahim et al. [47], RVF1 (777/5′-GAC TAC CAG TCA GCT CAT TAC C-3′/798) and RVF2 (1327/5′-TG TGA ACA ATA GGC ATT GG-3′/1309), were used to amplify a 551-base pair (bp) fragment of the G2 glycoprotein gene of the M segment. RT-PCR was performed using a Biorad DNA Engine thermocycler under the following conditions: cDNA synthesis at 50 °C, initial PCR activation and cDNA denaturation at 95 °C, 40 cycles at 94 °C for 45 s, 50 °C for 1 min, 72 °C for 1 min, and a final hold at 72 °C for 10 min. RT-PCR amplicons were visualized via gel electrophoresis using Apex Safe DNA gel strain (Genesee Scientific, CA, USA) on a 2% agarose gel. Bands were viewed using Spectroline UV transilluminator.

### 2.5. Virus Isolation on Vero Cells

Subconfluent African green monkey kidney (Vero-76) cells (ATCC, Manassas, VA, USA) seeded in 12-well culture plates were inoculated with 50 mL of mosquito pool supernatant and incubated for 1 h at 37 °C with 5% CO_2_ environment to allow virus adsorption into cells, prior to application of DMEM supplemented with 2% FBS, 0.37% sodium bicarbonate, 1% penicillin-streptomycin, and 1% Amphotericin B. Samples were then incubated as above for 14 days while monitoring daily for cytopathic effect (CPE). Samples indicating CPE were transferred to fresh Vero cells for confirmation, before supernatants were harvested and stored at −80 °C. Viral isolates were subsequently inactivated in a lysis buffer of QIAamp viral RNA mini kit and identified via RNA extraction and RT-PCR as above. For all *Aedes* spp. mosquito pools, and other samples that were RT-PCR-positive but not initially showing CPE, initial cell culture was blindly passaged at day 14 onto fresh cell monolayer and monitored for a further 14 days to assess the presence of detectable virus.

### 2.6. RVFV RNA Sequencing

All RVFV-positive samples (as assessed by cell culture isolation or molecular detection) were processed for Sanger sequencing. RVFV primers RVF1 and RVF2 as mentioned before were used to amplify the same G2 protein gene region (551 bp) of the M segment. Further primers RVFL10 (4237/5′-GGTGTTGTGTCATCATTG-3′/4254) and L2 (4730/5′-GTGTGAGCTAGADTTGCTTC-3′/4711) [48] were used to amplify a 494 bp fragment of the L segment. RT-PCR products were cleaned using a QIAquick PCR purification kit (Qiagen) according to the manufacturer recommendations and submitted for sequencing at the Virginia Tech Genomic Sequencing center [49]. Sequencing products were visualized using Sequence Scanner software (https://resource.thermofisher.com/page/WE28396_1/, accessed on 12 October 2024) and consensus sequences generated using Bioedit (https://api.semanticscholar.org/CorpusID:82421255, accessed on 12 October 2024). Consensus sequences were deposited in GenBank and given accession numbers (PQ753146—PQ753146), Table S1.

### 2.7. Data Analysis

To perform statistical analysis, mosquito data were entered into a comma-separated value (CSV) excel file and imported into Stata13. Generalized linear models (GLMs), Poisson, and negative binomial regression models for count data were used to assess the significance of differences in mosquito counts between variables—namely administrative districts, collection periods, and mosquito species. A difference was considered significant at the 95% confidence level (*p* < 0.05). Pooled minimum infection rates (MIRs) of mosquito species during a trapping period were estimated as a ratio of the number of infected pools in that period to the total number of tested species specimens (all sites combined) × 1000 [50]. For phylogenetic analysis, additional reference sequences for L and M segments of RVFV were selected from GenBank. Sequences were aligned using Clustal-W function in MEGA XI [51] software and the best fitting nucleotide substitution model was selected based on the lowest Bayesian Information Criterion (BIC) value. Maximum-likelihood phylogenetic trees were reconstructed using MEGA XI, Kimura 2 model with bootstrap support provided by 1000 iterations.

## 3. Results

### 3.1. Mosquito Distribution

A total of 14,815 mosquitoes, classified into five genera (*Culex*, *Anopheles*, *Mansonia*, *Coquillettidia*, and *Aedes*) and comprising at least 16 species, were collected in five locations during three temporal collection periods (Table 1). The majority of mosquitoes (82%) were caught via CDC light traps, followed by BG Pro (11%) and BG Sentinel traps (2%). Overall, 10, 14, 15, 13, and 13 mosquito species were identified in Bugesera, Rwamagana, Ngoma, Kirehe, and Kayonza districts, respectively.

Mosquito counts varied significantly between districts (X^2^ = 11.1, *p* = 0.02), with the highest abundance being found in Kayonza (36.2%), followed by Rwamagana (22.3%), Ngoma (17.7%), Bugesera (17.6%), and Kirehe (6.2%). Mosquito counts also varied significantly between collection periods (X^2^ = 6.1, *p* = 0.04), with the highest number collected during period 1, i.e., August–September 2021 (49.2%, n = 7292), followed by period 3, i.e., April–May 2022 (28.1%, n = 4164) and period 2, i.e., December 2021 (22.7%, n = 3359). The maximum average mosquito counts per trap per night were 638 for CDC light traps (period 1 in Kayonza), 30 for BG Pro (period 2 in Rwamagana), and 19 for BG Sentinel (period 2 in Bugesera) (Table S2).

### 3.2. Trapped Mosquito Species and Their Abundance

*Culex* genus was the most abundant (78.5%), with at least four species captured, followed by *Anopheles* genus (17.6%) with seven species, *Mansonia* (3.2%) with two species, and two species of *Coquillettidia* (0.5%). *Culex quinquefasciatus* was the most predominant species (X^2^ = 59.8, *p* < 0.0001), representing 72.7% of total counts, and displayed the highest prevalence across almost all sampling sites and trapping periods. Alongside *Culex* spp. (not able to be identified by species, 4.8%), *Cx. poicilipes* and *Cx. annulioris* in this genus were also captured but comprised less than 1% of total counts each. *Anopheles gambiae* (s.l.) (7.0%) was the second most predominant species, followed by *An. ziemanni* (5.5%), *An. maculipalpis* (2.8%), *Ma. africana* (2.0%), *An. squamosus* (1.4%), and *Ma. uniformis* (1.2%). Other species were less represented, with counts of less than 1%. *Aedes* spp. mosquitoes that are classified elsewhere as primary vectors of RVFV were caught in the fewest number during this study. Only 15 *Aedes* specimens (0.1%) were captured during the study, mostly in collection periods 2 and 3, and of these, seven were identified as *Ae. Aegypti*, and a further eight could not be identified by species.

### 3.3. RVFV Detection and Isolation

The 14,815 mosquitoes collected were tested as 765 mosquito pools, by both RT-PCR (detecting RVFV RNA) and virus isolation methods, with RVFV RNA detected in one pool of the 527 pools collected before the 2022 RVFV outbreak, and in five pools out of 238 pools collected during the 2022 outbreak (Table 2). The single RVFV-infected pool collected before the outbreak period comprised *Cx. quinquefasciatus* (0.4‰ MIR) trapped in Rwamagana district during collection period 2 (December 2021). The five positive pools collected during the 2022 Rwanda RVFV epizootic consisted of two pools of *Cx. quinquefasciatus* (0.6 ‰ MIR) collected in Kayonza and Rwamagana districts, two pools of *An*. *ziemanni* (5.7 ‰ MIR) collected in Ngoma district, and one pool of *An. gambiae* s.l. (7 ‰ MIR) collected in Kirehe district. During virus isolation, CPE was observed initially at days 8 and 11 post-inoculation, respectively, in the two RT-PCR-positive pools of *Cx. quinquefasciatus* collected during the outbreak, as mentioned above. RVFV was determined to be the arbovirus present in both isolates. No CPE was observed in any RT-PCR-negative sample. A second [blind] passage on Vero cells of all *Aedes* spp. mosquito pools and other RT-PCR positive/CPE-negative samples yielded negative results.

### 3.4. RVFV Sequencing and Phylogeny

**Figure 2:**
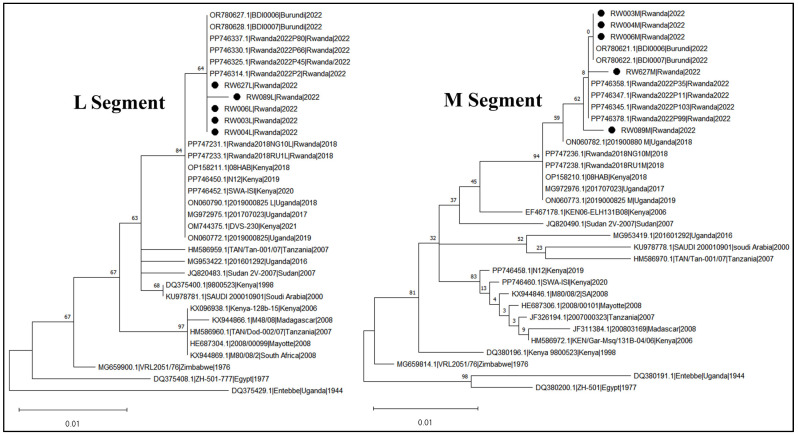
Maximum-likelihood phylogenetic tree based on RVFV L and M segments partial sequences. New sequences obtained in this study are marked with a black dot. The phylogenetic tree tips are labeled as accession, name, country, and year of specimen collection. Values below the node indicate the bootstrap support values (in %) for that node. The phylogenetic tree was reconstructed using MEGA 11 software [49].

Five RVFV partial genome sequences (comprising L and M segments) were recovered from the five pools of mosquitoes collected during the outbreak period. Two of these sequences were also obtained from RVFV isolated from cell culture. No sequence was obtained from the single positive pool of *Cx. quinquefasciatus* collected before the outbreak. The pairwise nucleotide identity comparison indicated that these viruses from mosquitoes were highly identical to each other, displaying only a maximum nucleotide difference of 0.003% in the M segment (sequenced fragment = 551 bp) and 0.002% in the L segment (sequenced fragment = 494 bp). The two cultured isolate genome sequences exhibited 100% similarity with sequences obtained directly from mosquito supernatant (thus, only the latter were presented). It was also observed that only *Cx. quinquefasciatus* isolate sequences displayed site mutations when compared with those recovered from *Anopheles* species pools. The maximum-likelihood phylogenetic trees reconstructed based on L and M segments of RVFV (Figure 2) showed that the viruses detected in mosquitoes during the 2022 Rwanda RVF outbreak were closely related to RVFV viruses isolated in livestock during the same outbreak in Rwanda. These isolates also clustered with viruses detected in the neighboring country of Burundi in the same year of 2022.

## 4. Discussion

The circulation of RVFV was first confirmed in Rwanda in 2012 [36,38] and subsequently caused two large epizootics that occurred in 2018 [39] and 2022 [40,52]. In 2022 alone, the outbreak unprecedently expanded in all the districts of the country and caused enormous socio-economic losses, including 22 human deaths, 1254 livestock abortions, and 516 animal deaths [52]. Efficient One Health-based interventions are thus essential in order to prevent or reduce the impact of future outbreaks. The determination of potential vector species and their infection rates is fundamental to any arbovirus risk assessment and outbreak prediction [41]. We report, for the first time in Rwanda, the results of RVFV entomological surveillance in mosquitoes collected from the eastern province before (August/September and December 2021) and during the 2022 Rwanda RVFV outbreak (April/May 2022). Both RT-PCR and cell culture isolation arboviral screening methods were used, and RVFV isolate sequences were obtained and analyzed.

In this study, a total of 14,815 mosquitoes were captured and tested as 765 mosquito pools for evidence of RVFV. Consistent with the previous studies in Rwanda [53,54], the catches comprised five mosquito genera: *Aedes*, *Culex*, *Anopheles*, *Mansonia* and *Coquillettidia*. A minimum of 17 mosquito species were identified, 11 of which were previously incriminated in RFV epizootics in different countries [12,21,23,25,26]. Based on mosquito vector classification according to the three criteria (natural infection, vector competence, and vector–host contact) proposed by Tantely et al. [25], five mosquito species captured during the current study qualify as potential vectors (only one criteria is validated), another five species as candidate vectors (two criteria are validated), and one as a vector (all three criteria are validated).

Five (2.1%) mosquito pools out of 238 pools collected during the 2022 Rwanda RVF outbreak tested positive to RVFV and were confirmed by sequencing. This proportion is comparable to that obtained in Kenya (71 of 3001 pools) [12] during the largest 2006/07 East Africa RVFV outbreak, and in Uganda [55] (3 of 296 pools) during an outbreak in Kabare district in 2016, but it was lower than that obtained during 2021 Madagascar outbreak (37/512) [26]. Among 527 pools collected in our study prior to the Rwandan outbreak, a single pool tested positive to RVFV. Although no RVFV sequence could be recovered from it, possibly because of low viral load generally common to inter-epidemic RVFV detections [56], our findings suggest that RVFV was circulating at least three months prior to the explosion of the 2022 RVF outbreak in Rwanda.

*Culex quinquefasciatus* was identified, not only as the most predominant species, but also as the species found infected with RVFV before and during the outbreak. In addition, *Cx. quinquefasciatus* was the only mosquito species, among the three species indicating RVFV-infection status, from which the virus was isolated by cell culture. These results suggest that this species may play a role in supporting the inter-epizootic circulation of the virus as well as the outbreak expansion. In nature, *Cx. quinquefasciatus*, which is also a vector of other important arboviruses such as West Nile, Zika, and chikungunya viruses [57,58,59], is found in a wide variety of habitats and can show a wide range of host feeding behavior including mammals, humans and birds [59]. High abundance of this species has been previously reported in different parts of Africa [60,61,62], and it has been implicated in many RVFV outbreaks elsewhere, including Kenya, Sudan and Mauritania [12,23,61,62]. In Sudan, RVFV was detected in the field-collected larvae and adult males, as well as in wings and legs of female *Cx. quinquefasciatus*, suggesting a natural transovarial transmission and virus dissemination within this taxon [62,63]. In laboratory studies, *Cx. quinquefasciatus* showed mixed results, depending on the geographic origin of the colonies used, with some experiments suggesting an incapacity to transmit RVFV [64,65], and others demonstrating low to moderate competence [66,67,68,69]. All these observations taken together suggest that *Cx. quinquefasciatus* is a vector of RVFV in Rwanda (three of the Tantely et al. criteria met [25]) and its role in the epidemiology of this disease and other arboviruses in the area deserves more investigation. In Rwanda, where small-scale cattle farming is predominant [35], and mostly practiced near the home, our investigation highlighted cattle manure (or dung) accumulation sites as one of the suitable breeding sites [70] for *Cx. quinquefasciatus* which overwhelmingly breeds in places rich in organic matter. We suggest this association between animals, vector, virus and humans constitutes an adequate condition for virus circulation with increased exposure to animals and humans. An efficient and integrated vector control program is thus recommended to interrupt this transmission cycle.

The detection of RVFV from three pools of malaria vectors namely *An. gambiae* s.l. (1 pool) and *An. ziemanni* (2 pools), which were second and third most abundant species, respectively, puts them onto the list of candidate vectors (two of the Tantely et al. criteria met [25]) of RVFV in Rwanda. *An. gambiae* s.l., one of the primary vectors of malaria parasites in Africa [58], was also found to be naturally infected by RVFV in Madagascar [26]. In Rwanda, two species of *An. gambiae* complex were previously reported: *An. gambiae* s.s. and *An. gambiae arabiensis*, with the latter being the most predominant [53]. Both immature and adult stages of *An. gambiae arabiensis* have been reported to harbor RVFV infection in the 2007/8 Sudan RVFV outbreak [62], though a dissemination study conducted by Moutailler et al. [69] reported the low susceptibility of *An. gambiae* to RVFV [69]. Since information on its vector competence for RVFV is not fully available, *An. gambiae* s.l only qualifies as an RVFV candidate vector thus far in Rwanda. *An. ziemanni*, which is categorized as a secondary malaria vector in Africa [58], has not, to our knowledge, previously been associated with RVFV infection in the field. However, this species used to belong to the *An. coustani* complex before being elevated to species level by Gillies and de Meillon in 1968 [71]. This group, with two species captured in our study (*An. coustani* and *An. ziemanni*), is mostly zoophilic and although *An. coustani* has been found to be naturally infected by RVFV in many countries [21,23,25,26] and shows susceptibility to infection in the laboratory [72], adequate evidence is still missing to confirm its vector competence to transmit the virus experimentally and thus, *An. ziemanni*, a group member, would be considered as a candidate vector in Rwanda.

Other species in our study that merit the “candidate vector” status (two of the three vector criteria met) include (i) *Culex poicilipes*, which feeds on livestock, birds, and humans [59], and has been found to be naturally infected in countries such as Senegal and Mauritania 1998/1999 [29], Kenya 2006 [12], and Sudan 2007 [62]. Its ability to transmit RVFV was repeatedly demonstrated in the laboratory [66,73]. However, following the collection of *Cx. poicilipes* in low numbers in our data, this species could only be designated as a candidate vector; (ii) two *Mansonia* species, namely *Ma. africana* and *Ma. uniformis*, which are also interesting candidate vectors of RVFV. Although their vectorial competence for RVFV is still undescribed, they have been found to be infected with the virus in several different countries [23,25] and a disseminated infection of 83.3% has been reported in *Ma. uniformis* collected during the 2006/07 Kenya RVF outbreak [27]. Our collected data indicated a sharp increase in *Ma. africana* and *Ma. uniformis* numbers during the outbreak, especially during mosquito trapping carried out in the wettest month of April 2022, in Bugesera, Kirehe, and Ngoma districts. A similar trend was observed in Baringo district of Kenya during the 2006 RVFV outbreak where these two *Mansonia* species, usually breeding in swampy areas, exhibited notable abundance, and alone they were capable to sustain the RVF outbreak in the area, contributing to 24% of infected mosquito pools [12].

Further mosquito species identified in our study, but in very low numbers, namely *Cx. annulioris*, *An. squamosus*, *An. funestus*, *An. coustani*, *An. rufipes*, and *Ae. aegypti*, would qualify as potential vectors (only one of the three vector criteria met) as they have been found to be naturally infected by RVFV in different parts of Africa [12,21,23,25,74,75], but have no well-described competency to transmit RVFV experimentally. *Aedes aegypti*, which is highly anthropophilic [59] and one of the most studied mosquito species due to its associations with major arboviruses such as dengue, chikungunya, Yellow fever and Zika virus [58], was found to be naturally infected by RVFV during the 2007 Sudan outbreak [62] and has shown the ability to transmit RVFV in the laboratory depending on the origin of the colony used [64,67,76,77]. However, this taxon is unlikely to be a major RVFV vector in the field as it has not been reported as a significant outbreak vector, despite its occurrence in many RVFV endemic areas [12,55,62,78].

The results from the phylogenetic analysis of RVFV in Rwandan mosquitoes, based on partial genome sequences of the L and M segments, indicated that the viruses isolated from mosquitoes in Rwanda were closely related to those isolated from livestock during the same RVFV outbreak that occurred in the country in 2022 [40,52]. These viruses, assigned to RVFV lineage C [40], also showed a close relatedness with viral strains detected in the neighboring country of Burundi in the same year of 2022, indicating an ongoing RVFV activity in the region. The tendency of accumulating mutations observed in the two geographically distant RVFV isolates from *Cx. quinquefasciatus*, absent in the three sequences detected from *Anopheles* spp. mosquito pools in Rwanda, need to be investigated with complete genome analysis in order to examine the eventual genomic change at finest resolution.

There were a number of limitations to the study including logistical challenges during the RVFV outbreak and sampling period, mainly associated with the COVID-19 pandemic, such as a lack of reliable supply of CO_2_ and dry ice. The lack of cold chain during mosquito identification could have affected the ability to isolate RVFV to some extent. Moreover, unexpected circumstances occurred during trapping periods, such as drought during collection period 2 [79]. Limited conclusions can therefore be drawn about the differences in mosquito distribution and abundance between districts or collection periods. In addition, the limited number of captured *Aedes* spp. specimens likely resulted from the sampling methods used. It has previously been observed that the profiles of trapped mosquito species significantly depend on the sampling location and the type of traps deployed [78]. Further studies using alternative sampling strategies are thus needed to determine the actual abundance and infection status of this mosquito genus in the region. In a previous randomized risk assessment study on Yellow fever virus conducted in Rwanda in 2012, trapped *Aedes* species (namely *Ae. aegypti*, *Ae. africanus*, *Ae. simpsoni*, *Ae. neoafricanus*, *Ae. mettallicus*, *Ae. Opock*, and *Ae*. (Finlay) sp.) comprised 9.6% of total counts [54]. Besides *Ae. aegypti* discussed above, it is noteworthy to mention that RVFV has been detected in *Ae. africanus* in Uganda (1956) and this taxon shall also be considered as potential vector in Rwanda. Another important factor that influenced the mosquito counts in this study was the coincidence of the trapping of collection period 3 during the 2022 outbreak with intensive insecticide spraying of livestock as one of measures deployed by the government to control the said RVFV outbreak. The insecticide repellency effect potentially interfered with mosquito collections that were being carried out near cattle shelters. There was no separation of engorged from unfed mosquitoes in the study; thus, there was the potential that host blood within blood-fed mosquitoes would obscure whether it was the mosquito or the vertebrate host that was infected with RVFV and affect conclusions of potential vector status. Future studies are advised to separate out engorged from replete mosquitoes to delineate the infective status of vector species. Additionally, while most blood meals here were assumed to be bovine due to the nature of sampling locations, host usage by mosquitoes can be confirmed via molecular analysis [27].

## 5. Conclusions

This entomological surveillance of RVFV in mosquitoes collected from the eastern province of Rwanda shows the existence of potential RVFV vectors in the country. Although there was no assessment of disseminated or transmissible RVFV infections, the findings reveal initial evidence for the incrimination of some mosquito species in the transmission of RVFV in the country. This study constitutes the first RVFV isolation from mosquitoes in Rwanda and adds to an existing accumulation of data on the continuous geographical expansion of RVFV endemicity in Africa, in which mosquitoes are among the key drivers. The findings also highlight the need for more studies to understand the role of each species in supporting the ecoepidemiology, transmission, and persistence of RVFV in Rwanda.

## Figures and Tables

**Table 1:** Summary of total mosquito counts per species, district, and collection period.

Mosquito Species	Bugesera			Rwamagana			Ngoma			Kirehe			Kayonza			Total	%	Pools Tested
	CP1	CP2	CP3	CP1	CP2	CP3	CP1	CP2	CP3	CP1	CP2	CP3	CP1	CP2	CP3			
*An. gambiae* s.l.	244	46	119	196	1	0	14	3	6	41	1	10	332	23	6	1042	7.0	47
*An.maculipalpis*	34	14	0	13	25	4	57	11	22	6	10	129	99	1	1	426	2.9	28
*An. squamosus*	3	2	2	5	74	12	46	5	32	5	13	0	1	0	5	205	1.4	17
*An. ziemanni*	0	16	157	42	139	13	96	144	106	1	3	58	10	18	13	816	5.5	47
*An. funestus*	0	0	0	0	0	1	44	34	0	1	0	0	0	3	4	87	0.6	10
*An. rufipes*	0	0	0	0	0	0	1	1	2	0	0	0	0	0	0	4	0.0	3
*An. coustani*	0	0	0	0	0	0	3	34	0	0	0	0	1	0	0	38	0.3	2
Total *Anopheles*	281	78	278	256	239	30	261	232	168	54	27	197	443	45	29	2618	17.6	154
*Cx. quinquefasc.*	119	405	1218	1195	1144	120	830	461	451	33	20	474	3108	434	757	10769	72.7	487
*Cx. poicilipes*	4	0	58	1	3	0	2	1	1	2	6	0	0	4	17	99	0.7	18
*Cx. annulioris*	1	0	0	4	26	1	0	8	0	0	9	0	0	2	0	51	0.3	8
*Cx.* spp. (NI)	0	0	54	134	13	0	0	6	0	0	0	0	469	28	13	717	4.8	42
Total *Culex*	124	405	1330	1334	1186	121	832	476	452	35	35	474	3577	468	787	11636	78.5	555
*Ma. africana*	7	12	40	17	32	6	17	28	56	0	0	83	0	1	0	299	2.0	19
*Ma. uniformis*	0	0	43	17	36	6	2	24	30	2	0	1	3	9	0	173	1.2	18
Total *Mansonia*	7	12	83	34	68	12	19	52	86	2	0	84	3	10	0	472	3.2	37
*Coq. maculipen.*	0	0	11	9	0	0	20	7	8	0	0	1	0	0	0	56	0.4	6
*Coq. aurites*	0	0	0	0	12	4	0	0	0	0	0	0	0	1	1	18	0.1	5
Total *Coq.*	0	0	11	9	12	4	20	7	8	0	0	1	0	1	1	74	0.5	11
*Ae. aegypti*	0	0	0	0	0	2	0	2	0	0	0	4	1	0	1	10	0.1	5
*Ae.* spp. (NI)	0	0	0	0	1	0	0	0	1	0	3	0	0	0	0	5	0.0	3
Total *Aedes*	0	0	0	0	1	2	0	2	1	0	3	4	1	0	1	15	0.1	8
Overall total	412	495	1702	1633	1506	169	1132	769	715	91	65	760	4024	524	818	14,815	100	765

CP = collection period. An. = *Anopheles*; Cx. = Culex; Ma. = *Mansonia*; Coq. maculipen. = *Coquillettidia maculipennis*; Cx. quinquefasc. = *Culex quinquefasciatus*; NI = could not be identified by species.

**Table 2:** Infected mosquito species and their minimum infection rate (MIR) estimated for the period of collection.

Collection Period	Mosquito Species	RT-PCR * Positive Pools	RVFV Isolated	Sequence Obtained **	Sequence ID	Site of Collection	No. Pools Tested	No. Mosq. Tested	MIR (‰)
Period 2	*Culex quinquefasciatus*	1	0	0	_	Rwamagana	138	2462	0.4
Period 3 (Outbreak)	*Anopheles* *ziemanni*	2	0	2	RW003 RW004	Ngoma	17	334	5.9
	*Anopheles* *gambiae* s.l.	1	0	1	RW006	Kirehe	10	141	7
	*Culex* *quinquefasciatus*	2	2	2 ***	RW089 RW627	Kayonza and Rwamagana	158	3020	0.6

* RT-PCR: Reverse transcription polymerase chain reaction; ** RVFV partial genome sequences obtained comprised L and M segments; *** RVFV partial genome sequences were obtained from two cell culture isolates as well as their uncultured counterparts; mosquito counts were aggregated per infected species and collection period to estimate the minimum infection rate (MIR).

## Data Availability

RVFV genome sequence data used in this study are available in GenBank.

## Supplementary Materials

The following supporting information can be downloaded at: https://www.mdpi.com/article/10.3390/pathogens14010047/s1, Table S1: Summary of RVFV genome partial sequences obtained from field- collected mosquitoes in Rwanda, and their associated Genbank accession numbers; Table S2: Average mosquito counts (and standard deviation) per trap per night depicted per mosquito genus, site and period of collection.
