# Supplementary material for: Identification of Potential Vectors and Detection of Rift Valley Fever Virus in Mosquitoes Collected Before and During the 2022 Outbreak in Rwanda
Source: Pathogens. 2025 Jan 8;14(1):47. doi: 10.3390/pathogens14010047 (PMC11768297; doi:10.3390/pathogens14010047)
Supplement: Supplementary file 1 [file pathogens-14-00047-s001.zip › pathogens-3407911-supplementary.pdf]

Supplementary materials

Table s1. Summary of RVFV genome partial sequences obtained from field collected mosquitoes in Rwanda and their associated GenBank accession numbers

Isolate name	Species	Date of collection	Site(district) of collection	Accession numbers		
				L segment	M segment	S segment
RW003	*Anopheles ziemanni*	26/04/2022	Ngoma	PQ753146	PQ753151	-
RW004	*Anopheles ziemanni*	27/04/2022	Ngoma	PQ753147	PQ753152	-
RW006	*Anopheles gambiae s.l.*	04/05/2022	Kirehe	PQ753148	PQ753153	-
RW089	*Culex quinquefasciatus*	26/05/2022	Kayonza	PQ753149	PQ753154	-
RW627	*Culex quinquefasciatus*	17/05/2022	Rwamagana	PQ753150	PQ753155	-

A dash (-) indicates that the segment was not included in sequencing.

Table s2. Average mosquito counts (and standard deviation) per trap per night depicted per genus, site and period of collection

**Collection period \ Site Name**		**Bugesera**			**Rwamagana**			**Ngoma**			**Kirehe**			**Kayonza**		
**Collection 1 (August – September 2021)**	**Mosquito genus**	**CDC LT**	**BG Pro**	**BG Sent.**	**CDC LT**	**BG Pro**	**BG Sent.**	**CDC LT**	**BG Pro**	**BG Sent.**	**CDC LT**	**BG Pro**	**BG Sent.**	**CDC LT**	**BG Pro**	**BG Sent.**
	Anopheles	11.5 (18.5)	22.2 (22.1)	9 (6.7)	17.5 (19.1)	9.5 (24)	9.5 (13.4)	45.8 (39.2)	0.75 (0.7)	0 (0)	7.6 (8.2)	1 (1.7)	0 (0)	62.1 (58.1)	8.75 (10.1)	0 (0)
	Culex	9.3 (9.1)	5 (6)	7 (4.7)	211.1 (326.3)	4.75 (5.8)	1.25 (2.5)	125.3 (128)	10 (7.8)	0 (0)	4.8 (3.2)	0.6 (0.5)	0.25 (0.5)	575.3 (371.9)	15.6 (27.5)	0 (0)
	Mansonia	1 (1.5)	0 (0)	0.25 (0.5)	4.8 (8.5)	0.6 (0.9)	0 (0)	5.3 (5.5)	0.2 (0.4)	0 (0)	0.3 (0.8)	0 (0)	0 (0)	0.5 (0.8)	0 (0)	0 (0)
	Coquillettidia	0 (0)	0 (0)	0 (0)	1.2 (1.8)	0.25 (0.7)	0 (0)	0.3 (0.5)	0 (0)	0 (0)	0 (0)	0 (0)	0 (0)	0 (0)	0 (0)	0 (0)
	Aedes	0 (0)	0 (0)	0 (0)	0 (0)	0 (0)	0 (0)	0 (0)	0 (0)	0 (0)	0 (0)	0 (0)	0 (0)	0 (0)	0 (0)	0 (0)
	**All Culicidae**	**21.8 (27.2)**	**27.2 (24.2)**	**16.2 (7.8)**	**235 (354)**	**14.1 (27.6)**	**6 (10.4)**	**176.8 (144.6)**	**11 (7.7)**	**0(0)**	**12 (11)**	**1.6 (1.8)**	**0.2 (0.5)**	**638 (418)**	**24.3 (34)**	**0(0)**
Collection 2 (December 2021)	*Anopheles*	3.2 (2.8)	6.75 (7.9)	1.25 (1.5)	16.8 (17.9)	8 (15.2)	0 (0)	29.2 (13.7)	2.1 (4.8)	1 (1.4)	4.5 (6.6)	0 (0)	0 (0)	3.2 (3.5)	3.1 (3.5)	0.2 (0.5)
	Culex	34.2 (44.7)	16.4 (12.7)	17.2 (14.9)	172.2 (257.3)	19.1 (18.2)	0 (0)	67.7 (45.2)	7.7 (15.2)	2.2 (2)	4.3 (7.7)	1 (1.3)	0.2 (0.5)	57.7 (65.2)	13.4 (20)	3.7 (4)
	Mansonia	1.2 (2)	0.4 (0.7)	0.5 (0.7)	7.7 (12.2)	2.7 (6.3)	0 (0)	6.7 (4.4)	1.2 (1.8)	0.5 (1)	0 (0)	0 (0)	0 (0)	1.5 (1.8)	0 (0)	0 (0)
	Coquilletidia	0(0)	0(0)	0(0)	0.8 (2)	0.9 (2.1)	0 (0)	1.2 (1.3)	0 (0)	0 (0)	0 (0)	0 (0)	0 (0)	0.2 (0.4)	0 (0)	0 (0)
	Aedes	0(0)	0(0)	0(0)	0 (0)	0 (0)	0.1 (0.5)	0 (0)	0.4 (0.5)	0 (0)	0.2 (0.4)	0.1 (0.3)	0.2 (0.5)	0 (0)	0 (0)	0.2 (0.5)
	**All Culicidae**	**38.5 (48)**	**23.5 (20.1)**	**19 (16.8)**	**210 (299)**	**30.7 (34.3)**	**0.2 (0.5)**	**105 (40)**	**11.3(21.3)**	**3.8 (3.3)**	**5.6 (12.9)**	**1.1 (1.2)**	**0.5 (0.6)**	**61 (69.5)**	**16.6 (22.9)**	**4.2 (3.5)**
Collection 3 (April – May 2022)	*Anopheles*	45.3 (73.2)	0.6 (1)	0.2 (0.5)	3.7 (3.6)	1 (2.4)	0 (0)	29 (12)	1.6 (2.2)	0 (0)	32.2 (63)	0.5 (0.7)	0 (0)	4.8 (3.2)	0 (0)	0 (0)
	Culex	196.3 (128.3)	11.7 (14.7)	14.5 (5.2)	11.2 (11.8)	3.6 (3.5)	6.2 (9.2)	61 (46.6)	9.4 (8.9)	2.7 (1.5)	57.8 (28.8)	13.5 (6.5)	4.7 (2)	128 (200.5)	2.6 (3.4)	0.2 (0.4)
	Mansonia	11 (13.7)	1.1 (1)	2 (2.8)	0.8 (1.2)	0.1 (0.3)	0.7 (0.9)	13 (15.6)	0.6 (0.7)	0.7 (0.9)	10.5 (15)	2.4 (4)	0.5 (1)	0(0)	0(0)	0(0)
	Coquilletidia	1.8 (4.5)	0 (0)	0 (0)	0 (0)	0(0)	0(0(	2 (3.5)	0.1 (0.3)	0 (0)	0.2 (0.4)	0 (0)	0 (0)	0(0)	0(0)	0(0)
	Aedes	0(0)	0(0)	0(0)	0 (0)	0.2 (0.7)	0 (0)	0.2 (0.4)	0 (0)	0 (0)	0 (0)	0 (0)	1 (1.4)	0 (0)	0(0)	0(0)
	**All Culicidae**	**254.5 (180.2)**	**13.5 (15.4)**	**16.7 (6.8)**	**16.1 (10.4)**	**5 (5.2)**	**17 (19.2)**	**105.1 (59.8)**	**11 (12)**	**3.5 (1.3)**	**100.6(92.2)**	**16.3(5.9)**	**6.2 (3)**	**132.8 (203)**	**2.3 (3.1)**	**0(0)**

CDC LT: Center for Disease Control light trap; BG Pro: Biogents Pro trap; BG Sent.: Biogents Sentinel trap
